# Targeting Multiple Receptors to Increase Checkpoint Blockade Efficacy

**DOI:** 10.3390/ijms20010158

**Published:** 2019-01-04

**Authors:** David J. Zahavi, Louis M. Weiner

**Affiliations:** 1 Department of Oncology, Lombardi Comprehensive Cancer Center, Georgetown University, Medical Center, 3800 Reservoir Rd NW, Washington, DC 20007, USA; djz8@georgetown.edu; 2 Lombardi Comprehensive Cancer Center Chair, Department of Oncology, Georgetown University Medical Center, 3970 Reservoir Road NW Research Building E501, Washington, DC 20015, USA

**Keywords:** immune checkpoint, combination therapy, receptor targets

## Abstract

Immune checkpoint blockade therapy is a powerful treatment strategy for many cancer types. Many patients will have limited responses to monotherapy targeted to a single immune checkpoint. Both inhibitory and stimulatory immune checkpoints continue to be discovered. Additionally, many receptors previously identified to play a role in tumor formation and progression are being found to have immunomodulatory components. The success of immunotherapy depends on maximizing pro-anti-tumor immunity while minimizing immunosuppressive signaling. Combining immune checkpoint targeted approaches with each other or with other receptor targets is a promising schema for future therapeutic regimen designs.

## 1. Introduction

The advent of immunotherapy has revolutionized cancer treatment. Specifically, the development of antibodies that target and block signaling through what have been termed “immune checkpoints” has led to promising clinical results in a variety of cancers. Antibodies against immune checkpoints such as cytotoxic T lymphocyte antigen 4 (CTLA-4), programmed death receptor 1 (PD-1), and programmed death-ligand 1 (PD-L1) have received FDA approval for the treatment of a growing number of solid tumors [1]. Despite the success of single-agent immune checkpoint blockade (ICB) therapy, clinical benefit has been limited to a minority of patients. Nevertheless, the promising anti-tumor activity of current ICB therapeutic regimens has led to continued interest in the development of newer checkpoint inhibitors and an exploration of other immunomodulatory agents. In addition, there is now focus on combining ICB with more conventional treatments and other immunotherapies to further improve clinical response rates and outcomes. The Society for Immunotherapy in Cancer (SITC) has convened the Combination Immunotherapy Task Force to address the promise and challenges of combining ICB with other therapies and the current status of these endeavors has been summarized elsewhere [2]. Various combinations of ICB are already under investigation, including combining ICB with more traditional chemotherapy and radiation [3]. There is a need for the design of rational immunotherapy based ICB combinations that maximize synergy by targeting other mechanisms important to the anti-tumor immune response such as immune cell priming, activation, and tumor mediated immunosuppression. It is therefore critical to understand the consequences of blocking the signaling of certain immune checkpoints or cell surface receptors whether alone or in combination. In this review we examine the currently approved and upcoming immune checkpoint inhibitors that will be used together with other therapies. We discuss the preclinical and clinical data supporting the use of immune checkpoint inhibitors in combination with each other and with other receptor targeted approaches. 

## 2. Checkpoint Blockade

Basic science research into the complexity of immune cell activation and regulation laid the foundation for ICB [4]. It is now appreciated that a variety of costimulatory and coinhibitory signals modulate immune cell responses to antigens. James Allison received the 2018 Nobel Prize in Medicine for discovering CTLA-4, the first coinhibitory receptor on T cells to be described. CTLA-4 is expressed on T helper and Treg cells and competes for binding of the ligands (CD80 and CD86) that provide a costimulatory signal when bound to CD28 expressed on T cells [5]. CTLA-4 is expressed on both CD4+ and CD8+ T cells and signals to inhibit priming of naïve CD4+ T cells, stimulate the immunosuppressive role of Tregs, and inhibit memory CD8+ T cell function [6,7,8]. Therefore, these results suggested that CTLA-4 blockade would directly and indirectly amplify T cell responses. ICB therapy based on CTLA-4 inhibition led to tumor regression in animal models [9]. Following these studies, monoclonal antibodies against CTLA-4 were swiftly developed and evaluated in clinical trials. Ipilimumab, a fully human immunoglobulin G1 (IgG1) anti-CTLA-4 antibody, became the first ICB therapy to receive FDA approval in 2011 after improving survival in melanoma patients in a large clinical trial [10]. Ipilimumab is the subject of a number of investigations and clinical trials in other cancer types as well [11]. Additionally, the fully human IgG2 anti-CTLA-4 antibody tremelimumab is in clinical trials as both a monotherapy and combination therapy with other ICB regimens [12].

PD-1 was originally described by Honju, another 2018 Nobel Prize recipient, as a receptor associated with the programmed death pathway in T cells, but was eventually found to play a role as a coinhibitory receptor that negatively regulates effector T cell function [13]. In fact, PD-1 is a key immune checkpoint receptor that is broadly expressed on activated CD8+ T cells, Tregs, and activated B cell and natural killer (NK) cells [14]. PD-1 signaling in tumor infiltrating lymphocytes (TILs) contributes to T cell exhaustion and tumors are known to upregulate the PD-1 ligand PD-L1 to exploit this pathway [15]. Since the PD-1 immune checkpoint signaling pathway directly plays a role in regulating immune responses of TILs in the tumor microenvironment, it is an ideal target for ICB therapy. The fully human IgG4 anti-PD-1 monoclonal antibody nivolumab received FDA approval for the treatment of melanoma in 2014 following improved outcomes in the CheckMate-037 clinical trial [16]. Since then, nivolumab and the similar humanized IgG4 monoclonal antibody pembrolizumab have gained a number of approvals for the treatment of a variety of cancers based on promising clinical trial results [17,18,19,20]. Other antibodies and inhibitors targeting either PD-1 (pidilizumab) or the ligands PD-L1 and PD-L2 (durvalumab and atezolizumab) are currently in clinical trials for many different indications [21]. The notable successes of ICB monotherapy regimens against the PD-1/PD-L1 axis have provided a framework that has become the foundation for combination strategies that will be discussed later.

The effectiveness of ICB led to the rapid expansion of the field of immune checkpoints and exploration of other inhibitory receptors found on immune cells. The therapeutic potential of targeting coinhibitory receptors that are members of the immunoglobulin superfamily, such as lymphocyte activation gene 3 (LAG3), T cell immunoglobulin and mucin domain-containing 3 (TIM3), T cell immunoglobulin and immunoreceptor tyrosine-based inhibitory motif domain (TIGIT), and V-domain Ig suppressor of T cell activation (VISTA), is under intense investigation. LAG3 is highly homologous to CD4 and has been found on activated T cells, Treg cells, NK cells, dendritic cells, and B cells [22]. Known LAG3 ligands include major histocompatibility complex class II (MHC-II) and the lectins galectin 3 and cell surface resident liver sinusoidal endothelial lectin (LSECtin) [23]. LAG3 is expressed both on Treg cells and exhausted TILs in the tumor microenvironment, suggesting a role in tumor immune evasion and that blockade can restore T cell anti-tumor function [24]. The LAG3-specific monoclonal antibody, relatilmab, is in ongoing clinical trials (e.g., NCT01968109 and NCT02061761). TIM3 is another T cell coinhibitory molecule that to date has had four ligands identified: Galectin-9, PtdSer, HMGB1, and CEACAM1 [25]. Expression of TIM3 on TILs is a marker of exhaustion and an inactive phenotype and may also function to abrogate NK cell responses [26,27]. Several anti-TIM3 monoclonal antibodies including TSR-022, Sym023, and MBG453 are in phase I clinical trials (NCT02817633, NCT03489343, and NCT02608268). TIGIT is a coinhibitory molecule that, along with similar proteins CD96 and CD112R, belongs to the poliovirus receptor (PVR)-like receptor family [28]. TIGIT and CD96 are both primarily expressed on T and NK cells and together with the costimulatory CD226 form an axis similar to the CD28/CTLA-4 pathway [29]. TIGIT blocking antibodies have entered clinical trials (NCT03119428) and there is strong preclinical evidence for blockade of CD96 [30]. Likewise, in vivo studies in mouse models indicate that blocking CD112R interactions on T cells or NK cells enhances anti-tumor responses [31,32]. B and T lymphocyte attenuator (BTLA) is yet another coinhibitory member of the immunoglobulin superfamily whose complex relationship with its ligand herpesvirus entry mediator (HVEM) has slowed its development as a therapeutic target [33]. VISTA, also known as PD-1H, is a member of the growing B7 family of checkpoint molecules. Antibodies targeting VISTA have entered clinical trial (NCT02671955), while the more recently discovered B7 family checkpoints are the subject of further investigation [34,35].

**Figure 1:**
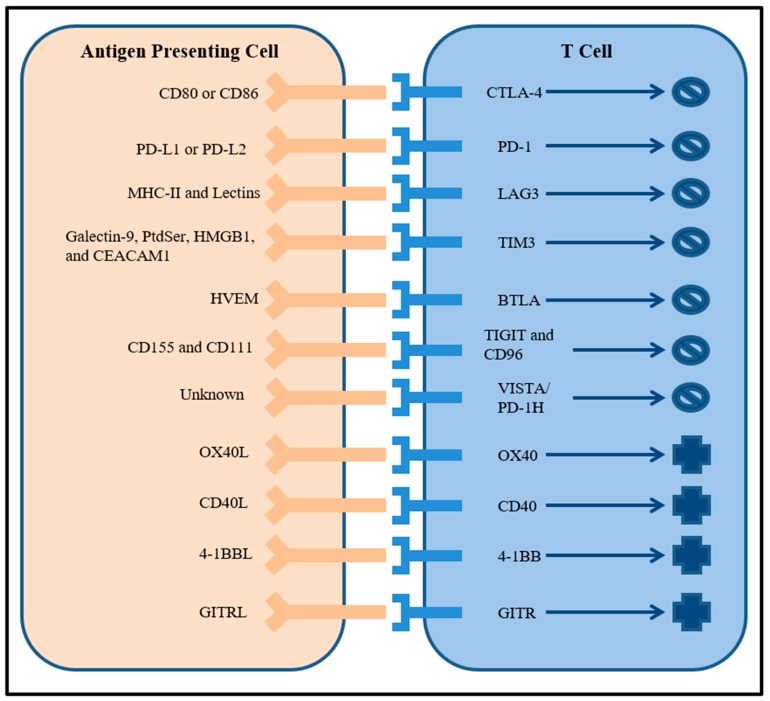
Cartoon of well characterized inhibitory and stimulatory immune checkpoint receptors on T cells and their ligands expressed by antigen presenting cells.

Costimulatory immune checkpoints are also being evaluated as potential therapeutic targets in addition to coinhibitory immune checkpoints. Molecules in the tumor necrosis factor (TNF) receptor superfamily such as OX40, CD40, 4-1BB, and glucocorticoid-induced TNF receptor (GITR) that are expressed on many immune cell types are prospective targets for agonistic antibodies [25]. For example, agonist antibodies to CD40 enhanced the function of antigen presenting cells and induced anti-tumor immunity [36]. Combining both activation of costimulatory receptors with inactivation of coinhibitory receptors represents a promising combination immunotherapy strategy that will be further explored in the next section. The large number of possible immune checkpoint receptor targets and their roles are symbolized in Figure 1.

## 3. Targeting Multiple Immune Checkpoints

Following their initial discoveries as critical negative regulators of T cell function, both CTLA-4 and PD-1 have had their signaling pathways extensively characterized. Although both checkpoints attenuate T cell activation, they do so by distinct mechanisms, in particular T cell subsets, and at different stages of T cell differentiation. Therefore, ICB therapy to each receptor will modulate T cell responses in a non-redundant manner, with anti-CTLA-4 primarily affecting the priming of T cells and anti-PD-1 enhancing T cell activity in the periphery and tumor microenvironment. Consequently, combining blockade of CTLA-4 and PD-1 is a potentially powerful synergistic therapeutic strategy. Initial exploration of the feasibility of dual checkpoint blockade in preclinical mouse models demonstrated that the combination of anti-CTLA-4 and anti-PD-1 enhanced anti-tumor activity compared to either regimen alone [37]. In 2015, the CheckMate-067 trial assessed the combination of ipilimumab and nivolumab in metastatic melanoma patients and concluded that combined therapy was effective and outperformed either agent alone in patients with PD-L1 negative tumors [38]. Follow-up reports from that study showed increased overall survival and progression-free survival in the patients who received dual therapy compared to monotherapy and, armed with additional successful results from CheckMate-069 [39], the FDA approved ipilimumab + nivolumab as the first ever immune checkpoint combination therapy. Since then, ipilimumab + nivolumab has been evaluated in additional cancer types and has received FDA approval for patients with advanced renal cell carcinoma and non-small cell lung cancer [1] while preclinical studies continue to determine the efficacy of this combination in other cancers such as BRCA1-mutated breast cancer [40]. While efficacious, combining ICB therapy is associated with increased risk for and severity of immune related adverse events associated with treatment that ultimately necessitates close monitoring of patients [41]. Overall, the efficacy, tolerability, and safety of ipilimumab + nivolumab has led to a large number of clinical trials testing various combinations of anti-CTLA-4 and anti-PD-1/anti-PD-L1 antibodies.

The established anti-tumor activity of targeting the PD-1 signaling axis as a monotherapy in a variety of cancers coupled with a favorable toxicity profile when compared to CTLA-4 inhibition provided robust justification for selecting anti-PD-1 antibodies as the backbone of combinatorial strategies. Preclinical studies of additional checkpoint inhibitors have revealed other possible therapeutic synergies. Thus, many of the above-mentioned clinical trials for new checkpoint modulating antibodies include experimental arms that assess their efficacy in combination with some form of anti-PD-1. The rationale for inclusion of these combinations has been based on mechanistic data from in vivo studies. Both LAG3 and TIM3 are frequently found to be co-expressed with PD-1 on inactive TILs and dual ICB managed to effectively reverse tumor specific T cell anergy more than monotherapy in preclinical models [42]. An antibody directed to LAG3 is in clinical trial combined with nivolumab for the treatment of glioblastoma (NCT02658981) while the antagonist LAG-3 mAb (LAG525) is in a phase I/II study as a combination treatment with PD-1 inhibition in solid tumors (NCT02460224). Likewise, for TIM3, there are upcoming phase II clinical trials combining anti-TIM3 with anti-PD-1 in liver cancer (NCT03680508) and other advanced solid malignancies (NCT03744468). TIGIT is also upregulated in tumor specific TILs and dual blockade with anti-PD-1 enhanced anti-tumor immunity [43]. Again, these observations suggested that TIGIT and PD-1 blockade act in an additive manner to augment T cell function. While antibodies to CD96 have yet to enter the clinic, an inhibitor of another PVR-like receptor family member, CD112R, has begun a phase I clinical trial combined with nivolumab (NCT03667716). VISTA and PD-1 have also been shown to have separate inhibitory effects on T cells [44]. Further study of the B7 family of immune checkpoints will ideally identify synergistic mechanisms of action with anti-PD-1 that can guide future combination therapy design.

Uniting antibodies that block inhibitory receptors with agonistic antibodies that activate stimulatory receptors are complementary strategies that should lead to a high degree of synergy for potentiating the anti-tumor immune response. Several preclinical studies have indicated that this is the case [45]. A costimulatory receptor found on T cells and NK cells, 4-1BB, improves both T cell and NK cell anti-tumor effects upon activation [46,47]. Phase I and II clinical trials to evaluate 4-1BB agonists in combination with anti-PD-1 therapy have either begun (NCT02253992) or are already completed (NCT02179918). Activating GITR stimulates the proliferation of both CD4+ and CD8+ T cells and activation reverses Treg cell-mediated suppression leading to an enhanced anti-tumor immune response [48]. A GITR agonist antibody in combination with nivolumab proved to be successful in a clinical trial for patients with advanced solid tumors [49]. Many other combinations featuring GITR agonist antibodies are in ongoing clinical trials (NCT03126110 and NCT03277352). Whereas GITR is constitutively expressed, OX40 becomes expressed only on activated T cells [50]. Therefore, while no OX40 agonists are being tested as a monotherapy, the combination of OX40 activating antibodies with inhibitory checkpoint blockade has shown promise and is the subject of a number of clinical trials (NCT01714739 and NCT01750580).

Several additional receptors have emerged as potential important immune checkpoint targets. Adenosine signaling through the A2aR on immune cells in the tumor microenvironment has been described as increasing immunosuppression. Inhibiting A2aR not only enhanced TIL function in a murine cancer model, it modulated T cell expression of other coinhibitory checkpoints which suggested possible synergy with anti-PD-1 therapy [51]. Combining an A2aR antagonist with targeting the PD-1 axis is under investigation in both solid tumors and hematological malignancies (NCT03207867). Similarly, evidence from in vivo models indicates that nuclear receptor subfamily 2, group F (Nr2f6) acts as an immune checkpoint that also regulates the expression of other checkpoints and combining their blockade enhances TIL function [52]. As more immune checkpoint receptors are discovered and characterized, efficient combinatorial therapies that maximize their synergistic potential will be designed and tested.

## 4. Combining Checkpoint Blockade with Other Receptor Targets

The clinical success of ICB and its combinations has led to the development of combinatorial strategies that expand to include other receptor targeted therapies. ICB stimulates immune cells directly; however, there are many other immunosuppressive mechanisms at play in the tumor microenvironment. Approaches that primarily address these additional mechanisms such as immunosuppressive cytokine blockade, angiogenesis inhibition, and enhancement of tumor immunogenicity will be particularly powerful when joined with ICB.

For example, TGFβ is a cytokine that not only directly promotes tumor growth but also extrinsically suppresses anti-tumor immunity in the tumor microenvironment [53]. Inhibitors of the TGFβ receptor restored anti-tumor immune responses in a manner independent of immune checkpoint status and synergized with anti-PD-1 therapy to lead to durable responses in mouse models [54]. The cytokine colony stimulating factor-1 (CSF-1) is implicated in the recruitment of immunosuppressive myeloid derived suppressor cells (MDSCs) to the tumor [55]. In murine models, an anti-CSF-1 receptor antibody could prevent MDSC recruitment and relieved suppression of anti-CTLA-4 ICB [56]. One of the most well studied cytokines known to have important functions in the tumor microenvironment is vascular endothelial growth factor (VEGF). Angiogenesis driven by VEGF signaling is believed to play a role in solid tumor progression and anti-angiogenic therapies are now being considered also as anti-tumor immune modulators [57]. VEGF modifies the anti-tumor immune response at several levels including the prevention of TIL trafficking and promotion of immunosuppressive Treg and MDSC subsets [58]. The benefits of targeting VEGF signaling through its receptors VEGFR1 and VEGFR2 in combination with checkpoint blockade has been under careful investigation as not all anti-VEGFR tyrosine kinase inhibitors (TKIs) are capable of promoting an immune response [59]. That said, the multi-TKI axitinib potentiated checkpoint blockade in preclinical models [60] and is now in clinical trials (e.g., NCT03172754). In addition to TKIs, antibodies directed towards VEGFR2 have been proven to enhance T cell infiltration into tumors which synergizes well with anti-PD-1 therapy in mice [61]. The anti-VEGFR2 antibody ramucirumab has entered clinical trials in combination with nivolumab for many different cancer types (NCT03527108 and NCT02999295).

There is also accumulating evidence that TKIs directed towards either EGFR or HER2 in cancers that overexpress those receptors may exert immunomodulatory effects that influence ICB. In a spontaneous EGFR+ tumor model, an EGFR TKI had an immunomodulatory effect in addition to its direct cytotoxic effect on tumor cells and increased the number of TILs. This therapeutic effect was further augmented by combination with anti-CTLA-4 [62]. Further study suggests a correlation between EGFR activation and a signature of immunosuppression highlighted by the upregulation of PD-1, PD-L1, CTLA-4, and tumor-promoting inflammatory cytokines. Clinical trials for the combination of EGFR TKIs and nivolumab in EGFR mutant lung cancer have delivered promising results [63]. Likewise, anti-HER2 antibodies were found to synergize with a multitude of ICB regimens in preclinical models [64]. Moreover, some receptors are overexpressed in certain cancer types and play unique roles in facilitating an immunosuppressive tumor microenvironment. Pancreatic ductal adenocarcinoma (PDAC) is typically unresponsive to most immunotherapies due to its particularly immunosuppressive microenvironment. PDAC cells and the cancer associated fibroblasts supporting them express the cholecystokinin receptor (CCKR) of which signaling directly promotes tumor growth and TIL-limiting fibrosis [65]. A CCKR antagonist was able to reduce fibrosis, lead to an influx of TILs, and boost ICB therapy efficacy in murine models of PDAC [65].

Finally, several receptors on cells of the innate immune system are yet another potential target for combination with checkpoint blockade. Toll-like receptors (TLRs) are found on dendritic cells, NK cells, and macrophages and activation of TLRs leads to the production of factors that stimulate inflammatory and pro-immunity pathways. Agonists of TLRs have previously been investigated for their anti-cancer properties; however their complex role in both pro- and anti-tumor signaling has complicated their therapeutic development [66]. Nevertheless, immunostimulating TLR9 agonists are in various phases of clinical trial in combination with PD-1 inhibitors (NCT03618641, NCT03326752, and NCT03445533). Stem cell factor receptor (c-KIT) is a known oncogene but has more recently been described as a modulator of MDSC function [67]. Anti-c-KIT monoclonal antibody treatment abrogated immunosuppressive MDSC function and enhanced ICB efficacy in murine tumor models [68]. A group of receptor tyrosine kinases (RTKs) called TAM found on dendritic cells and NK cells are being considered as innate immune system checkpoints whose blockade may propagate adaptive immunity and complement conventional ICB therapy [69]. The TKI sitravatinib, which inhibits a broad spectrum of RTKs including not only VEGFR and c-KIT but also the TAM family when added to nivolumab, is in phase II clinical trials for clear cell renal cell and urothelial carcinoma (NCT03680521 and NCT03606174). Another receptor-ligand pair that may be relevant as an innate immune checkpoint is the CD47-SIRPα axis. CD47, also known as the “don’t eat me” signal, is expressed on normal cells to prevent macrophage engulfment but is often overexpressed on malignant cells to engage its receptor SIRPα found on anti-tumor macrophages and neutrophils in order to prevent phagocytic function [70]. Beyond its effects on phagocytes, anti-CD47 treatment was found to modulate the tumor microenvironment to be more permissive for anti-tumor T cells in preclinical models of head and neck squamous cell carcinoma [71]. Similarly, an antagonistic antibody to the receptor SIRPα enhanced the anti-tumor activity of macrophages and neutrophils against a wide variety of cancer types [72]. Taken together, these results suggested that inhibiting the CD47-SIRPα axis was a promising candidate for combination therapy meant to simultaneously stimulate both the innate and adaptive immune responses. Inhibitors of the CD47-SIRPα signaling axis have just begun to enter clinical trials in order to evaluate their efficacy in combination with PD-1 blockade (NCT03530683 and NCT03013218).

## 5. Conclusions

ICB has been a boon for the treatment of many deadly cancers and has significantly improved clinical outcomes. Unfortunately, only a subset of patients and cancer types currently show favorable response rates to ICB, in part due to the varying immunogenicity of different tumors and immunosuppressive tumor microenvironments. Combination immune checkpoint therapy is a promising method for extending these positive results. Numerous immune checkpoints have since been identified, and are in varying stages of pre-clinical and clinical development. Furthermore, combining ICB with other receptor targets can help mediate stronger anti-tumor immune responses by inhibiting other pathways of tumor mediated immunosuppression. The success of ICB regimens will greatly depend on multi-pronged approaches that tackle the variety of immunosuppressive mechanisms using innovative ICB combinations [73].
